# Cryptococcal Meningoencephalitis in an Immunocompetent Patient: A Case Report

**DOI:** 10.7759/cureus.100500

**Published:** 2025-12-31

**Authors:** Kailie A McGee, Pardis A Zarandi, Sanjna Tripathy, Sherly Mathew

**Affiliations:** 1 Internal Medicine, Baylor Scott & White All Saints Medical Center, Fort Worth, USA

**Keywords:** atypical headache, cryptococcal meningitis (cm), cryptococcal meningoencephalitis, cryptococcal neoformans/gattii cryptococcal meningoencephalitis, fungal lumbar puncture, fungal meningitis, immunocompetent adult

## Abstract

Cryptococcal meningoencephalitis (CM) is an infection of the brain and spinal cord caused by Cryptococcus species, an opportunistic fungus. Immunocompetent patients who present with subtle or nonspecific symptoms may experience a delayed diagnosis of CM. We present a case of a 25-year-old immunocompetent woman who arrived with severe headache, fever, and mild meningeal symptoms. Initial infectious and imaging studies were unrevealing, and lumbar puncture revealed an elevated opening pressure with normal cerebrospinal fluid indices. Despite lacking conventional risk factors, she was found to have a positive Cryptococcus antigen in serum on two repeated tests. Her workup did not identify an underlying immunodeficiency. A later careful re-evaluation of the history revealed that she had exposure to backyard poultry. She improved gradually with antifungal therapy and was discharged on long-term fluconazole. This case illustrates the diagnostic complexity of CM in patients without overt immunosuppression and emphasizes the importance of considering fungal meningitis in the differential diagnosis of persistent or atypical headaches. Increased awareness of these presentations is critical, as timely recognition and early antifungal treatment remain essential to reducing morbidity and preventing neurologic complications.

## Introduction

Meningitis refers to inflammation of the protective membranes surrounding the brain and spinal cord. Cryptococcal meningoencephalitis (CM) is a fungal opportunistic infection that is most commonly seen in immunocompromised individuals, usually those with HIV/AIDS, organ transplantation, or during the use of immunosuppressive medications. Every year, an estimated 152,000 cases of CM occur worldwide in people with HIV, and 112,000 deaths occur [1]. Although the global burden continues to impact immunosuppressed populations disproportionately, sporadic cases in healthy individuals have been increasingly reported. The most common causative species, *Cryptococcus neoformans*, is typically associated with immunocompromised hosts, whereas *Cryptococcus gattii *has been more frequently identified in patients without known immune deficits. Studies from other countries, like Australia and British Columbia, report *C. gattii *death rates ranging from 13% to 33% [1]. In summary, these two species differ in host predilections, environmental niches, and clinical manifestations.

Presentation of immunocompetent patients with Cryptococcus species may deviate from classic meningitic symptoms (fever, nuchal rigidity, headache, photophobia) and instead present as seemingly milder symptoms such as persistent headaches [2]. Patients with fungal meningitis may appear less acutely ill despite endorsing symptoms commonly associated with meningitis. Although symptoms may appear mild in the beginning, disease burden can quickly become extremely severe. A study examined four immunocompetent patients with no obvious underlying risk factors and their presenting symptoms, most of which were nonspecific and generally unremarkable [3]. The pathogenesis of seemingly healthy patients who contract cryptococcal species is not fully understood, but the presence of polymorphisms, such as in Dectin-2 and granulocyte-macrophage colony-stimulating factor, that disrupt macrophage function is a consideration [4]. This case highlights the diagnostic complexity of CM in immunocompetent patients and how the insidious onset of this disease often causes diagnostic delays, especially in resource-constrained areas. Timely consideration of fungal meningitis remains vital, even in otherwise healthy and young patients.

## Case presentation

A 25-year-old immunocompetent female with a BMI of 51.52 (historically and upon presentation), microcytic anemia, and vertigo presented to the emergency department (ED) with a chief complaint of 10/10 generalized headache. Other associated symptoms included generalized weakness, nausea, and fatigue. A week prior to presentation, she was having chest pain that radiated to her back and neck. She noticed a fever peaking at 102°F three days prior to arrival. In the ED, she described the headache as dull and achy, and it worsened when lying flat or walking. On presentation, she denied diarrhea, shortness of breath, orthopnea, paroxysmal nocturnal dyspnea, lower extremity edema, appetite changes, confusion, brain fog, or recent travel. She endorsed mild neck stiffness and normal oral intake of both solid and liquid.

Of note, she met sepsis criteria with a temperature of 102.5°F and was tachycardic with a heart rate of 110 beats per minute. She was tachypneic with a respiratory rate of 23 breaths per minute. The physical examination revealed mild neck stiffness, pain with flexion, and negative Kernig and Brudzinski signs. Her laboratory results in the ED were notable for a normal lactate level, a negative comprehensive viral panel, and a negative urinalysis. Her chest X-ray (CXR) showed central vascular congestion, and her CT head and CT abdomen and pelvis were negative for acute processes. A lumbar puncture (LP) was attempted but was unable to be completed due to the patient's body habitus, so an interventional radiology-guided LP was ordered along with an echocardiogram (ECHO). She was admitted for sepsis of unknown origin.

Once admitted, the infectious disease (ID) service was consulted, and a variety of tests were sent out, including HIV, meningitis viral panel, herpes simplex virus (HSV), John Cunningham (JC) virus, Cytomegalovirus (CMV)/Epstein-Barr virus (EBV), and syphilis, all of which were negative. A lumbar puncture was successfully completed and demonstrated normal cell counts with an opening pressure of 29 cmH_2_O (Table 1). She was found to have a positive cryptococcal blood antigen test with negative titers, due to high levels of antibody (Table 2). The repeat to confirm was also positive. Her IgA, IgG, and IgM immunoglobulins were within normal limits. Of note, her typhus fever antigen tests were also found to be positive, with IgG positive at 1:128 and IgM levels >1:1024 (Table 3). Her antibodies also demonstrated IgG at 1:64 and IgM >1:256. An MRI of the brain was completed on the patient, and it was negative, along with her ECHO.

**Table 1:** Cerebrospinal fluid (CSF) laboratory results.

Tests	Patient's results	Reference ranges
Clarity, CSF	Clear	Clear
Color, CSF	Colorless	Straw, colorless
Red blood cell count, CSF (per μL)	0	<1
Total nucleated cell count, CSF (per μL)	4	0-5
Volume, CSF (mL)	10.5	-
Xanthochromia, CSF	Absent	Absent
Glucose, CSF (mg/dL)	102	40-70
Protein, CSF (mg/dL)	34	15-45
Culture, CSF	No growth at 4 days	No growth
*Cryptococcus neoformans/gattii*, CSF	Not detected	Not detected
Cryptococcal antigen, CSF	Negative	Negative

**Table 2:** Fungal blood tests.

Tests	Patient's results	Reference ranges
Initial cryptococcal antigen	Positive	Negative
Initial cryptococcal antigen titer	1:2	No antigen detected
Repeat cryptococcal antigen	Positive	Negative
Repeat cryptococcal antigen titer	1:10	No antigen detected

**Table 3:** Typhus test results.

Tests	Patient's results	Reference ranges
Typhus fever antibodies titer, IgG	1:64	<1:64
Typhus fever antibodies titer, IgM	≥1:256	<1:64
Initial typhus fever, IgG	1:128	<1:64
Initial typhus fever, IgM	>1:1024	<1:64
Repeat typhus fever, IgG	Detected	Undetected
Repeat typhus fever, IgM	Detected	Undetected

As the treatment progressed, the regimen included vancomycin 1250 mg IV q8h, ceftriaxone 2 g IV, fluconazole 400 mg daily, and dexamethasone 10 mg IV for four days; her symptoms slowly improved. She still occasionally complained of a waxing and waning headache along with mild nausea. She was discharged with six months of fluconazole 400 mg daily and seven days of doxycycline for the positive typhus serologies. During her stay, and after an extensive workup, we were unable to identify risk factors for her acquiring this disease. The only potential source identified was her father's mention that they own multiple chickens in their backyard. The patient was discharged with fluconazole 400 mg once daily for six months and was recommended to have close outpatient follow-up. Currently, we are unable to obtain outpatient medical records to determine whether she followed up as recommended.

## Discussion

Cryptococcal meningoencephalitis remains a devastating global health concern, with almost all reported cases in humans being caused by the following two main strains: *Cryptococcus neoformans *species complex and its emerging sister species, *Cryptococcus gattii*. *C. gattii* has been recognized as an endemic pathogen in Australia, but in the 1990s, it gained attention for emerging into British Columbia, Canada, and the Pacific Northwest, demonstrating its ability to expand and adapt [5].

*C. gattii* has been found in multiple studies to have a predilection for immunocompetent patients [5]. Recently, however, some studies have reported that *C. neoformans *plays a more significant role than previously hypothesized [3]. One prospective study in Vietnam included 57 participants, 81% of whom had no underlying diseases, and found that *Cryptococcus neoformans *accounted for 70% of infections [6].

It is unclear why these organisms have a striking neurotropism. One theory discusses the idea that the elevated dopamine levels in the central nervous system (CNS) are used as a substrate to synthesize melanin, a major virulence factor [7]. According to a study, this would also explain why dopamine-rich areas are frequently affected by cryptococcal lesions. A second theory is that the production of D-mannitol by these organisms may lead to its accumulation in brain tissue, causing edema and thereby inhibiting phagocytic functions [8].

Common risk factors include HIV, AIDS, and organ transplantation, with more uncommon causes like prolonged glucocorticoid treatment, genetic disorders, liver disease, and sarcoidosis [9-11]. This means that although patients may be labeled as "immunocompetent," several studies have demonstrated subtle predisposing risk factors, which are the most likely cause in our patient’s case. One of the most well-established is the presence of anti-granulocyte-macrophage colony-stimulating factor (GM-CSF) autoantibodies, which impair macrophage activation. One study found a notable correlation between otherwise healthy HIV negative patients and a positive high-titer autoantibody test, with or without the development of associated pulmonary alveolar proteinosis (PAP) [12].

As mentioned earlier, CM in immunocompetent patients often presents insidiously and is more common in younger patients. Healthy individuals exhibit more meningitis-related symptoms, visual symptoms, and auditory symptoms [13]. They also had an increased time from symptom onset to diagnosis [13]. Immunocompetent patients, such as the one described in this report, are more symptomatic due to a stronger inflammatory and immune response. Due to their nonspecific symptoms, they are often misdiagnosed and are not correctly diagnosed until much later. Our patient stated that prior to her arrival in the ED, she had seen two different physicians - one diagnosed her with migraines, and the other suggested an infectious cause with an unknown source. A similar case of a 40-year-old female with chronic headaches and no neurologic findings took months to be diagnosed with this life-threatening disease [14]. This case illustrates the diagnostic challenge posed by CM in immunocompetent patients and underscores the importance of considering it in the differential diagnosis of select patients with persistent, progressive, or atypical headache presentations, even in the absence of traditional risk factors.

## Conclusions

In this case, a young adult who appeared immunocompetent developed cryptococcal meningoencephalitis, suggesting that environmental exposures and/or subtle host factors can contribute to disease. Her nonspecific symptoms and delayed diagnosis illustrate the diagnostic challenges CM can pose outside traditional high-risk populations. Clinicians should maintain a high index of suspicion for fungal meningitis in patients with persistent or atypical headaches, regardless of immune status. Early recognition, timely lumbar puncture, and prompt antifungal therapy are critical for managing similar cases. This case reinforces the need for broader awareness of CM presentations and the importance of considering environmental exposures, infection with potentially more virulent Cryptococcus species, and subtle host risk factors such as anti-GM-CSF autoantibodies or genetic polymorphisms affecting innate immunity (e.g., Dectin-2), even in patients without overt immunosuppression.

## References

[1] (2025). Cryptococcosis facts and stats. https://www.cdc.gov/cryptococcosis/data-research/index.html.

[2] Correa K, Craver S, Sandhu A (2021). An uncommon presentation of cryptococcal meningitis in an immunocompetent patient: a case report. Clin Pract Cases Emerg Med.

[3] Stack M, Hiles J, Valinetz E, Gupta SK, Butt S, Schneider JG (2023). Cryptococcal meningitis in young, immunocompetent patients: a single-center retrospective case series and review of the literature. Open Forum Infect Dis.

[4] Onyishi CU, May RC (2022). Human immune polymorphisms associated with the risk of cryptococcal disease. Immunology.

[5] Chen SC, Meyer W, Sorrell TC (2014). Cryptococcus gattii infections. Clin Microbiol Rev.

[6] Chau TT, Mai NH, Phu NH (2010). A prospective descriptive study of cryptococcal meningitis in HIV uninfected patients in Vietnam - high prevalence of Cryptococcus neoformans var grubii in the absence of underlying disease. BMC Infect Dis.

[7] Kwon-Chung KJ, Rhodes JC (1986). Encapsulation and melanin formation as indicators of virulence in Cryptococcus neoformans. Infect Immun.

[8] Wong B, Perfect JR, Beggs S, Wright KA (1990). Production of the hexitol D-mannitol by Cryptococcus neoformans in vitro and in rabbits with experimental meningitis. Infect Immun.

[9] Spec A, Raval K, Powderly WG (2016). End-stage liver disease is a strong predictor of early mortality in cryptococcosis. Open Forum Infect Dis.

[10] Prevel R, Guillotin V, Imbert S, Blanco P, Delhaes L, Duffau P (2022). Central nervous system cryptococcosis in patients with sarcoidosis: comparison with non-sarcoidosis patients and review of potential pathophysiological mechanisms. Front Med (Lausanne).

[11] Ruschel MA, Thapa B (2023). Cryptococcal meningitis. StatPearls [Internet].

[12] Rosen LB, Freeman AF, Yang LM (2013). Anti-GM-CSF autoantibodies in patients with cryptococcal meningitis. J Immunol.

[13] Makakala MC (2023). Cryptococcal meningitis in immunocompetent patients. Int J Clin Stud Med Case Rep.

[14] Nakahira S, Yamamoto M, Wood T (2025). An unusual clinical presentation of cryptococcal meningitis: the importance of a detailed history and physical. IDCases.

